# AI-driven prediction of severe respiratory sequelae in COVID-19 patients

**DOI:** 10.1080/07853890.2025.2598914

**Published:** 2025-12-12

**Authors:** Yao Li, Lihua Liao, Xianyue Hu, Lufang Huang, Lin Zhang

**Affiliations:** Liuzhou People’s Hospital, Liuzhou, Guangxi, P. R. China

**Keywords:** COVID-19, respiratory sequelae, unsupervised machine learning, convolutional neural networks, precision medicine

## Abstract

**Background:**

In coronavirus disease 2019 (COVID-19) patients, respiratory sequelae are common complications that significantly impact health outcomes. Hence, early identification of patients at risk is essential for improving prognosis and care.

**Materials and Methods:**

We enrolled 516 COVID-19 patients and applied K-means algorithms to cluster them into subtypes based on clinical characteristics and risk profiles. ResNet-50 was employed to analyze and extract features from chest X-rays, accurately identifying COVID-19-related lesions. The extracted imaging data were integrated with clinical data to develop a predictive model aimed at stratifying post-COVID-19 patients by risk and identifying those likely to develop severe respiratory sequelae.

**Results:**

We identified two distinct COVID-19 subtypes, one of which was associated with severe respiratory sequelae. The convolutional neural networks (CNNs) accurately detected COVID-19-related lesions on chest X-rays. The predictive model showed excellent subtype discriminative ability, achieving an area under the curve (AUC) of 0.949 and 0.958 in the training and validation cohorts, respectively.

**Conclusions:**

Our AI-driven predictive model demonstrates strong potential for the early identification of respiratory sequelae in COVID-19 patients. By applying the K-means algorithm to cluster patients based on clinical characteristics, in combination with feature extraction from chest X-rays using the ResNet-50 deep learning model, we accurately stratified patients by their risk of severe respiratory outcomes. However, to evaluate its performance in clinical settings, further validation using larger, independent datasets is essential to confirm the model’s reliability and generalizability across diverse populations.

## 1. Introduction

The coronavirus disease 2019 (COVID-19) pandemic has emerged as the most lethal infection of the twenty-first century [1]. Research and healthcare efforts primarily focused on addressing acute COVID-19 cases during the pandemic [2]. However, as the outbreak is subsiding, more attention is now focused on its long-term effects as well as understanding the prevalence and management of post-COVID-19 syndrome.

Post-COVID-19 syndrome encompasses several symptoms and health issues experienced by individuals in the post-acute COVID-19 phase. These symptoms may persist for months after the initial infection [3]. Due to ‘long-COVID-19′ or ‘post-COVID-19′ syndrome manifestations, estimating the prevalence of post-COVID-19 syndrome remains challenging; the reported rates range from 2.3% to 51% [4]. Respiratory sequelae, a significant cluster of post-COVID-19 conditions, encompass respiratory symptoms and underlying pulmonary disease [5]. In post-COVID-19 patients, lung damage remains a significant concern. Studies have shown that fibrosis and reduced pulmonary function can impair quality of life for months or even years [6]. Respiratory sequelae are commonly observed, with challenges such as reduced exercise tolerance and persistent lung damage continuing even after recovery from the acute phase [7]. Survivors of severe cases, particularly those who required intensive care, frequently report decreased exercise tolerance, chronic breathlessness and other physical limitations [8,9].

As subsets of artificial intelligence, unsupervised machine learning algorithms (UMLAs) and convolutional neural networks (CNNs) have made significant advancements in medicine, particularly in areas such as computer-assisted screening, decision support and precision diagnostics [10–13]. Our study aimed to explore these AI tools to achieve effective risk stratification of respiratory sequelae severity in COVID-19 patients. By using the K-means algorithm, a popular unsupervised machine learning algorithm, our objective was to identify subtypes of COVID-19 patients into subtypes who were at a higher risk of severe respiratory outcomes. Subsequently, we developed a predictive model by integrating ResNet-50, a CNN-based deep learning (DL) technique commonly used for image analysis, with clinical data and chest X-rays (CXRs). This model aims to assist clinicians in proactively managing COVID-19 patients, facilitating early interventions to potentially prevent the progression of severe respiratory sequelae and improve patient outcomes.

While numerous studies related to COVID-19 patients currently exist, the majority focus on epidemiological analyses during the pandemic and predictions of severe cases [14,15]. Research on the management of sequelae in COVID-19 patients remains comparatively scarce. To address this gap, our research utilized UMLAs and CNNs to perform risk stratification for COVID − 19 patients, enabling the early identification of those who may develop sequelae and providing guidance for clinicians. By extracting deep learning features from CNNs and merging them with clinical variables, we created a multidimensional dataset that captures both imaging-derived biological signatures and established clinical risk factors. This hybrid framework addresses a critical gap in existing literature, where predictive models often rely solely on either clinical parameters or imaging data, limiting their comprehensiveness.

Moreover, the combination of UMLAs and CNNs for this specific purpose has received relatively little attention. UMLAs aim to discover the inherent structures and patterns in data without explicit labels. By combining unsupervised machine learning with CNNs, we can potentially uncover hidden relationships and features in medical data and build more accurate predictive models.

By bridging these methodological and clinical gaps, our work provides a clinically actionable tool for pre-emptive identification of high-risk patients, facilitating targeted interventions to mitigate long-term respiratory morbidity.

In this study, we first applied UMLAs to cluster clinical data from COVID-19 patients, followed by the implementation of CNN-based deep learning on the CXRs. By integrating patients’ clinical characteristics with deep learning-derived features, we ultimately developed a predictive model to identify COVID-19 patients at risk of severe respiratory sequelae. This model enables risk stratification of COVID-19 patients, guiding clinicians in delivering early interventions to high-risk individuals and mitigating the progression of potential severe respiratory sequelae.

## 2. Research significance

Despite increasing attention to post-COVID-19 syndrome, critical gaps remain in understanding and managing severe respiratory sequelae. Current research on predictive tools for respiratory complications in COVID-19 survivors is limited, with few models specifically designed to stratify risk for severe long-term respiratory outcomes. Existing approaches often rely on isolated data sources, such as clinical parameters alone or imaging findings in isolation, which may overlook the complex interplay between clinical characteristics and physiological changes in the lungs. Additionally, the heterogeneity of post-COVID-19 manifestations makes it challenging to identify high-risk subgroups, hindering targeted early intervention.

Our study developed a comprehensive approach that bridges clinical and imaging insights. By first identifying patient subtypes based on shared clinical features and then integrating these subtypes with detailed lung imaging analysis, the research moves beyond single-dimensional assessments. This dual focus on clinical patterns and lung imaging characteristics allows for a more nuanced understanding of which patients are at greater risk of severe respiratory sequelae, filling the gap in tailored risk stratification tools.

**Figure 1.**
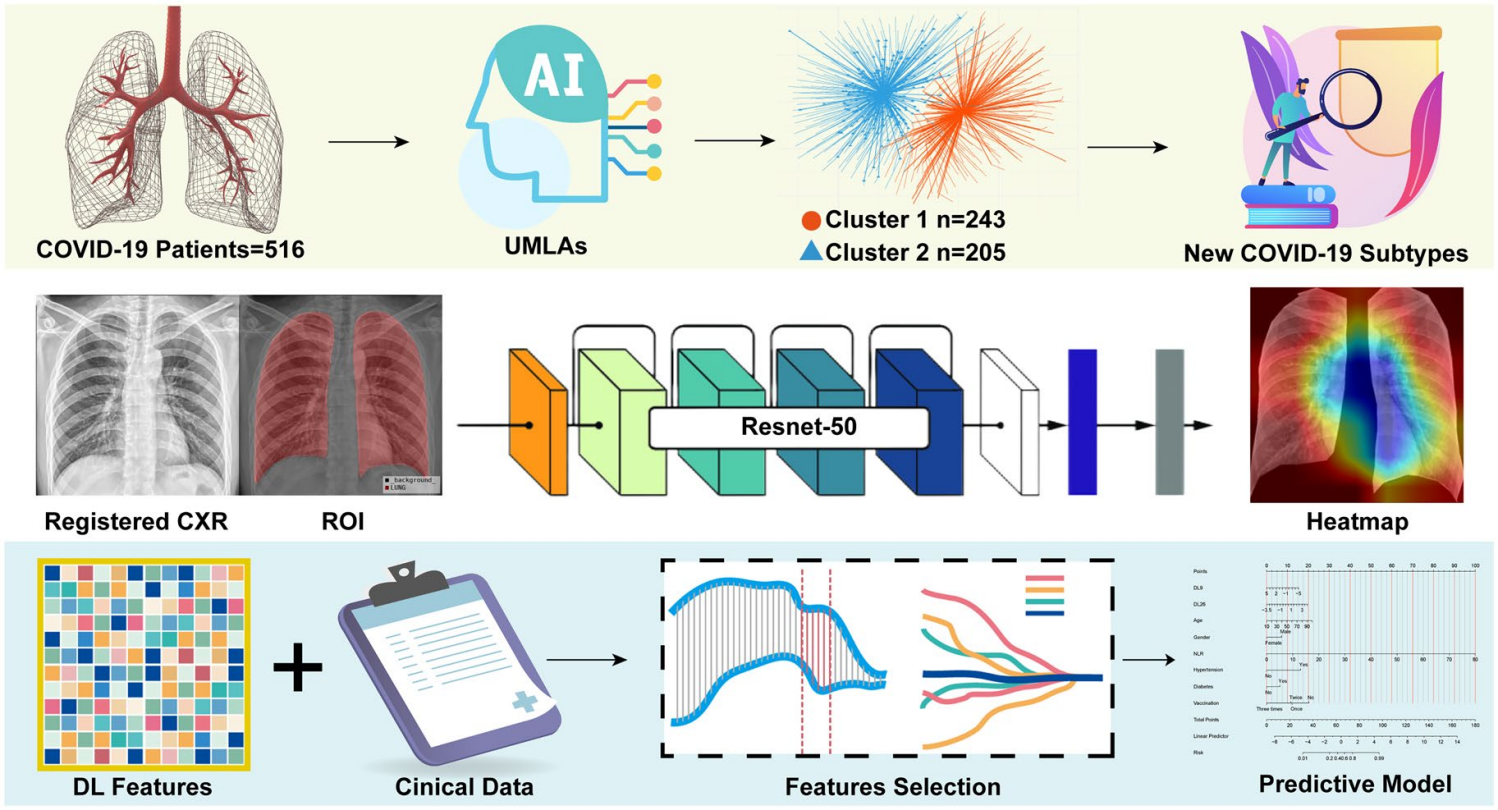
The graphical abstract of this study. We used unsupervised machine learning algorithms to classify COVID-19 patients based on similar clinical characteristics. UMLAs successfully identified COVID-19 patient subtypes at risk for severe respiratory sequelae clinically. Then, we utilized ResNet-50 to annotate and extract chest X-ray images of COVID-19 patients. Using clinical and imaging data, we established a precise predictive model for early identification of respiratory sequelae in COVID-19 patients. DL: Deep learning; ROI: Region of interest; UMLAs: Unsupervised machine learning algorithms.

This study lies in its potential to enhance clinical practice for post-COVID-19 care. By establishing a framework that combines clinical profiling with lung imaging analysis, the study provides a foundation for early identification of high-risk patients. This, in turn, supports timely interventions to mitigate long-term respiratory damage, improve patient quality of life and optimize the allocation of healthcare resources. Figure 1 presents a graphical abstract of the study.

## 3. Materials and methods

### 3.1. Patients

Our study was conducted as per the Declaration of Helsinki and received approval from the Ethics Committee of Liuzhou People’s Hospital (KY2024-058-01). Written informed consent was obtained from the individuals for the publication of any potentially identifiable images or data included in this article. Additionally, clinical data and CXRs were anonymized to remove all identifiable information.

We retrospectively analyzed the clinical data of COVID-19 patients admitted to Liuzhou People’s Hospital’s fever clinic between December 2022 and March 2023. The patient data included CXR images, baseline clinical characteristics, routine blood test results and vaccination profiles. The inclusion criteria were: (a) all confirmed COVID-19 patients aged 18 years or older, (b) patients with complete clinical data and (c) patients with follow-up data available from 3 months to 1 year post-diagnosis. The exclusion criteria were as follows: (a) death during the acute phase of COVID-19 infection, (b) incomplete clinical data records and (c) poor-quality chest X-ray (CXR) images unsuitable for analysis. Finally, we included 516 patients in the study.

### 3.2. Identification of COVID-19 subtypes using UMLAs

Predictor and outcome variables were collected from the clinical data for assessing the clustering accuracy, respectively. The predictor variables comprised gender, age, body mass index (BMI), neutrophil-to-lymphocyte ratio (NLR), platelet count, haemoglobin level, comorbidities and smoking as well as vaccination status respectively. The follow-up data were utilized to derive outcome variables and included respiratory symptoms (breathlessness, erratic breathing and persistent cough) as well as underlying pulmonary diseases like lung fibrosis and thromboembolic disease.

The K-means algorithm was employed to cluster COVID-19 patients and identify distinct subgroups by minimizing intragroup variation. Renowned for its simplicity and computational efficiency, the K-means algorithm is particularly suitable for unsupervised machine learning tasks. Its straightforward implementation has made it a widely used tool in various clinical studies. We employed the K-means++ initialization algorithm to avoid poor clustering results from random centroid selection. The algorithm proceeded as follows: (a) Randomly selected the first centroid from the dataset.; (b) Computed the squared Euclidean distance ($D(x{)}^{2}$ between each data point and the nearest existing centroid.; (c) The cluster centroids were recomputed based on the assigned points, selected subsequent centroids probabilistically, where points with larger $D(x{)}^{2}$ between each data point and the nearest existing centroid; and (d) Steps 2–3 were repeated until the centroids stabilized between iterations or Maximum iterations ($T_{\text{max}}=300$) is reached [16]. We utilized the scale function from the ‘factoextra’ package in R version 4.2.1 for predictor variables’ standardization [17]. Moreover, ‘fpc’ software helped to calculate the optimal number of clusters, and the silhouette coefficient was calculated to assess clustering efficiency [18]. The silhouette coefficient measure evaluates the unsupervised learning model’s performance by calculating how well each data point fits into its assigned cluster.

K-means clustering was used to divide the patients into two prediction-factor-based clusters. Subsequently, the two clusters’ outcome variable differences were analyzed to verify the K-means clustering accuracy.

### 3.3. DL Analysis and interpretability

The CXRs obtained upon patient admission were analyzed to characterize the initial radiographic presentation of the cohort. Among the 516 patients included in the study, 267 (51.7%) exhibited abnormal findings on their initial CXR. The spectrum of observed abnormalities was dominated by ground-glass opacities, present in 197 patients (38.2%), followed by consolidation in 96 patients (18.6%) and interstitial thickening in 64 patients (12.4%). Before conditioning the DL model, the COVID-19 patients’ CXR images were preprocessed. Original CXR images were transformed into a Joint Photographic Experts Group format from Communications in Medicine. Furthermore, all images were resized using B-spline interpolation to a 256 × 256 matrix grid and normalized to 256 gray levels for image data standardization. This B-spline interpolation was chosen due to its ability to preserve the sharpness of the image while avoiding distortion, making it suitable for medical image processing where high-quality details are critical. LabelMe (version 5.0.1) tool helped to segment manually-segregated anonymized CXR images [19], with the region of interest (ROI) encompassing the entire lung.

**Figure 2.**
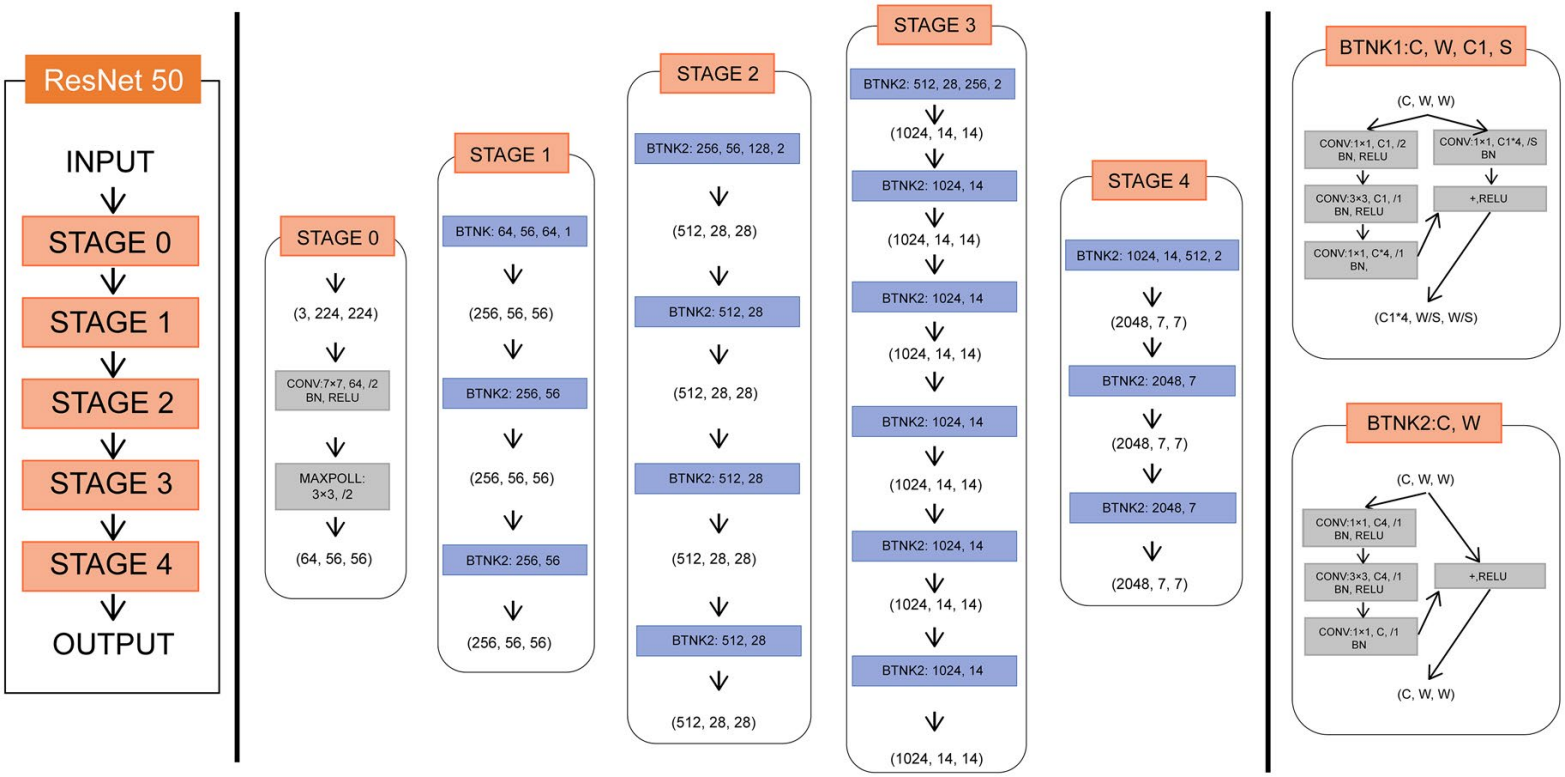
Framework of ResNet-50.

Our DL model utilized the Residual Convolutional Neural Network (ResNet50) model due to its ease of training, enhanced feature reusability and efficient parameter utilization. These traits are particularly advantageous for large-scale datasets and iterative model training [20]. Figure 2 displays the framework of ResNet50. We categorized 516 patients into distinct COVID-19 subgroups based on the K-means cluster results. The preprocessed CXR images were split in an 8:2 ratio into training and validation sets, respectively. The training phase involved the use of an Adam optimizer with a learning rate of 0.001 and a batch size of 64. Utilizing early stopping with L2 regularization helped to mitigate the risk of overfitting. We assessed the model’s efficacy by the Receiver Operating Characteristic (ROC) curve. Each patient’s CXR was handled as a distinct input throughout the DL procedure. Upon its completion, DL features were extracted from the fully connected layer within the CNNs.

The preprocessed CXRs were split in an 8:2 ratio into training and validation sets, and the training phase involved the use of an Adam optimizer with a learning rate of 0.001 and adjusted using a step decay schedule. A batch size of 64 was employed to balance memory efficiency and gradient stability during training. The model was trained for 50 epochs. Binary cross-entropy loss was used, suitable for the classification tasks in our model. Early stopping, combined with L2 regularization and a dropout rate of 0.5, was employed to penalize large weights and prevent overfitting.

For visualization, gradient-weighted class activation mapping (Grad-CAM) was employed to enhance the transparency of the model’s decision-making process [21]. Grad-CAM highlights key regions in the target classification image by generating a class activation map using gradient data from the final convolutional layer of the CNNs. To ensure the accuracy and clinical relevance of the Grad-CAM results, two experienced radiologists independently reviewed the highlighted regions.

### 3.4. Construction of ensemble predictive model

Combining clinical and DL features created an ensemble dataset. It was then divided into training and validation cohorts in an 8:2 ratio. The training cohort used the least absolute shrinkage and selection operator (LASSO) logistic multivariate regression analysis to identify independent risk factors. This training dataset helped to develop a multivariate logistic regression model, and its coefficients were used for constructing a nomogram. The nomogram provided a visual representation of the predicted probabilities by mapping the logistic regression coefficients onto a 0–100 scale [22]. Its performance was also evaluated using the ROC and calibration curves, with the area under the ROC curve (AUC) ranging from 0.5 (no discrimination) to 1 (perfect discrimination). Additionally, we used decision curve analysis (DCA) to establish the net benefit threshold for prediction.

### 3.5. Statistical analysis

We used SPSS version 26.0, R version 4.2.1, and GraphPad Prism 9 for statistical analyses. Clinical data were presented as mean (SD). We employed the Student’s t-test, Mann-Whitney U test, or chi-square test, depending on the type of data, to identify differences between the two clusters. Statistical significance was set at *p* < 0.05.

## 4. Results

### 4.1. UMLA results

**Figure 3.**
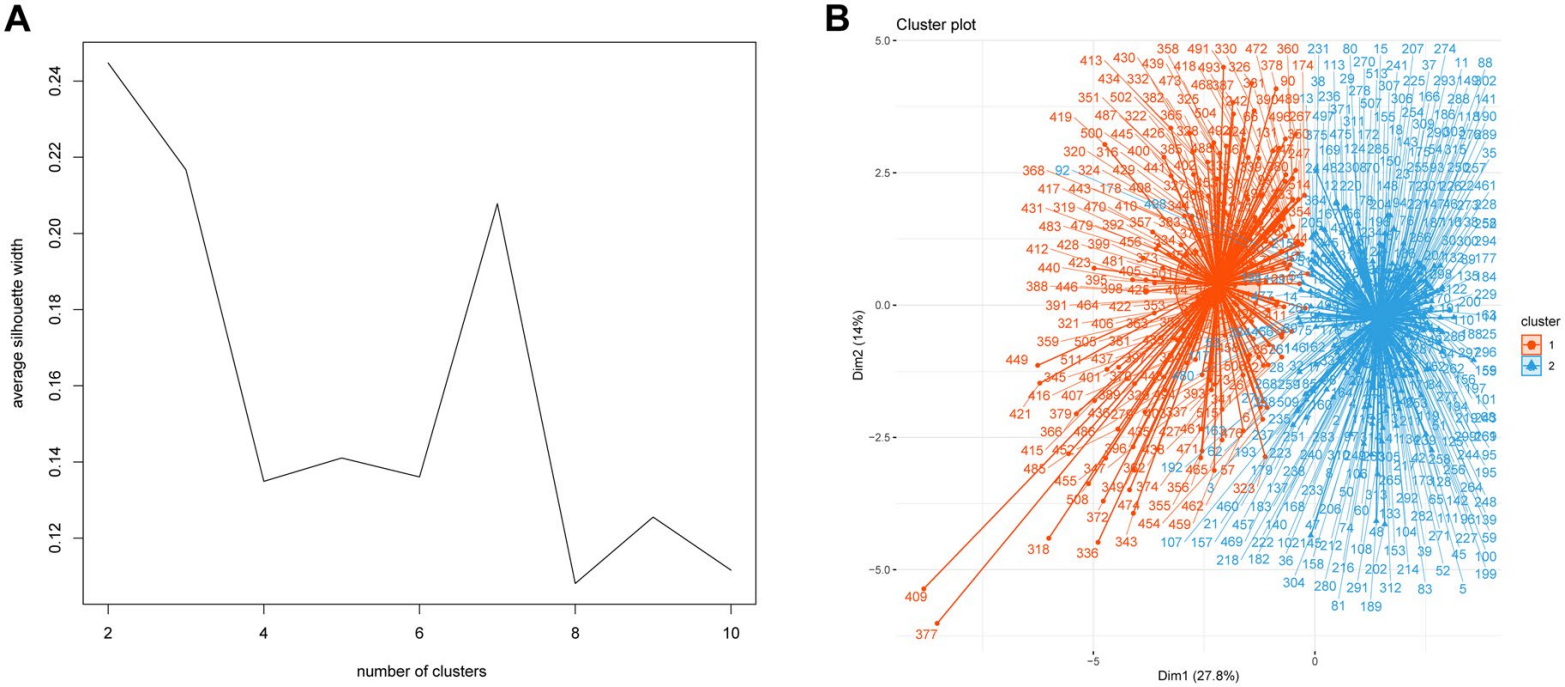
Result of unsupervised machine learning. (A) Optimal clustering number of the K-means clustering algorithm was determined by Silhouette coefficient (SC). The peak of the curve is the best value for the Silhouette coefficient (Y-axis); the best number of clusters was equal to 2 (X–axis). (B) Scatter plots of patients’ clinical data. Scatter points on the graph represent each patient. The K-means algorithm divides patients into two clusters. The red scatter represents cluster 1 and the blue scatter represents cluster 2.

Figure 3A displays the optimal number of clusters determined by the K-means algorithm, where the silhouette coefficient curve’s peak indicates that two clusters are the ideal quantity. Based on this algorithm, the clinical data were categorized into two clusters, Clusters 1 and 2, respectively (Figure 3B). Table 1 presents the predictor variables’ K-means clustering results.

**Table 1.** Predictor variables of the study patients by clusters.

Characteristic	Cluster		p-value
	1, *N* = 243	2, *N* = 205	
Age	73.50 (63.25, 80.00)	42.00 (31.00, 65.25)	<0.001
BMI	23.77 (21.04, 26.00)	22.63 (20.44, 25.35)	0.017
NLR	12.11 (6.87, 22.20)	3.95 (2.06, 7.97)	<0.001
Platelet	202.50 (149.00, 251.50)	204.00 (167.75, 255.00)	0.160
HGB	123.00 (105.75, 136.00)	136.00 (125.75, 146.00)	<0.001
Gender			<0.001
Female	73 (35.78%)	199 (63.78%)	
Male	131 (64.22%)	113 (36.22%)	
Smoking			0.264
No	184 (90.20%)	290 (92.95%)	
Yes	20 (9.80%)	22 (7.05%)	
Hypertension			<0.001
No	77 (37.75%)	269 (86.22%)	
Yes	127 (62.25%)	43 (13.78%)	
Diabetes			<0.001
No	122 (59.80%)	280 (89.74%)	
Yes	82 (40.20%)	32 (10.26%)	
COPD			<0.001
No	147 (72.06%)	289 (92.63%)	
Yes	57 (27.94%)	23 (7.37%)	
Vaccination			<0.001
No	110 (53.92%)	38 (12.18%)	
Once	28 (13.73%)	24 (7.69%)	
Twice	21 (10.29%)	38 (12.18%)	
Three times	45 (22.06%)	212 (67.95%)	

BMI: Body mass index; COPD: Chronic obstructive pulmonary disease; NLR: Neutrophil-to-lymphocyte ratio; HGB: haemoglobin.

Cluster 1 predominantly consisted of older individuals with higher clinical severity indicators. This cluster had a significantly higher mean age of 73.50 years (IQR: 63.25, 80.00) compared to Cluster 2, which had a mean age of 42.00 years (IQR: 31.00, 65.25) (*p* < 0.001). Furthermore, Cluster 1 exhibited higher values in BMI, NLR and haemoglobin levels, suggesting more severe physiological distress. Notably, Cluster 1 also had a significantly higher prevalence of hypertension (62.25% vs. 13.78%, *p* < 0.001), diabetes (40.20% vs. 10.26%, *p* < 0.001) and chronic obstructive pulmonary disease (COPD) (27.94% vs. 7.37%, *p* < 0.001). In terms of gender distribution, Cluster 1 had a higher proportion of males compared to Cluster 2 (64.22% vs. 36.22%, *p* < 0.001), indicating a significant gender imbalance between the two clusters.

### 4.2. Comparison of outcome variables between the two clusters

Table 2 compares the outcome variables between the two clusters. Cluster 1 exhibited significantly higher rates of respiratory complications compared to Cluster 2, suggesting a more severe clinical profile. Specifically, Cluster 1 had a significantly higher prevalence of breathlessness (35.29% vs. 8.65%, *p* < 0.001), erratic breathing (31.37% vs. 3.85%, *p* < 0.001) and persistent cough (40.20% vs. 11.22%, *p* < 0.001). Additionally, Cluster 1 was also more likely to suffer from pulmonary fibrosis (23.04% vs. 4.81%, *p* < 0.001) and pulmonary embolism (14.22% vs. 3.53%, *p* < 0.001).

**Table 2.** Comparison of outcome variables between two clusters.

Characteristic	Cluster		p-value
	1, *N* = 224	2, *N* = 293	
Breathlessness			<0.001
No	132 (64.71%)	285 (91.35%)	
Yes	72 (35.29%)	27 (8.65%)	
Erratic Breathing			<0.001
No	140 (68.63%)	300 (96.15%)	
Yes	64 (31.37%)	12 (3.85%)	
Persistent Cough			<0.001
No	122 (59.80%)	277 (88.78%)	
Yes	82 (40.20%)	35 (11.22%)	
Pulmonary Fibrosis			<0.001
No	157 (76.96%)	297 (95.19%)	
Yes	47 (23.04%)	15 (4.81%)	
Pulmonary Embolism			<0.001
No	175 (85.78%)	301 (96.47%)	
Yes	29 (14.22%)	11 (3.53%)	

These significant differences in outcome variables between the clusters further validate the accuracy and clinical relevance of the K-means clustering approach used in this study. The findings indicate that Cluster 1 represents a subgroup of COVID-19 patients at higher risk for severe respiratory sequelae, highlighting the potential utility of this clustering method for identifying high-risk patients for targeted interventions.

### 4.3. Performance of the DL model

**Figure 4.**
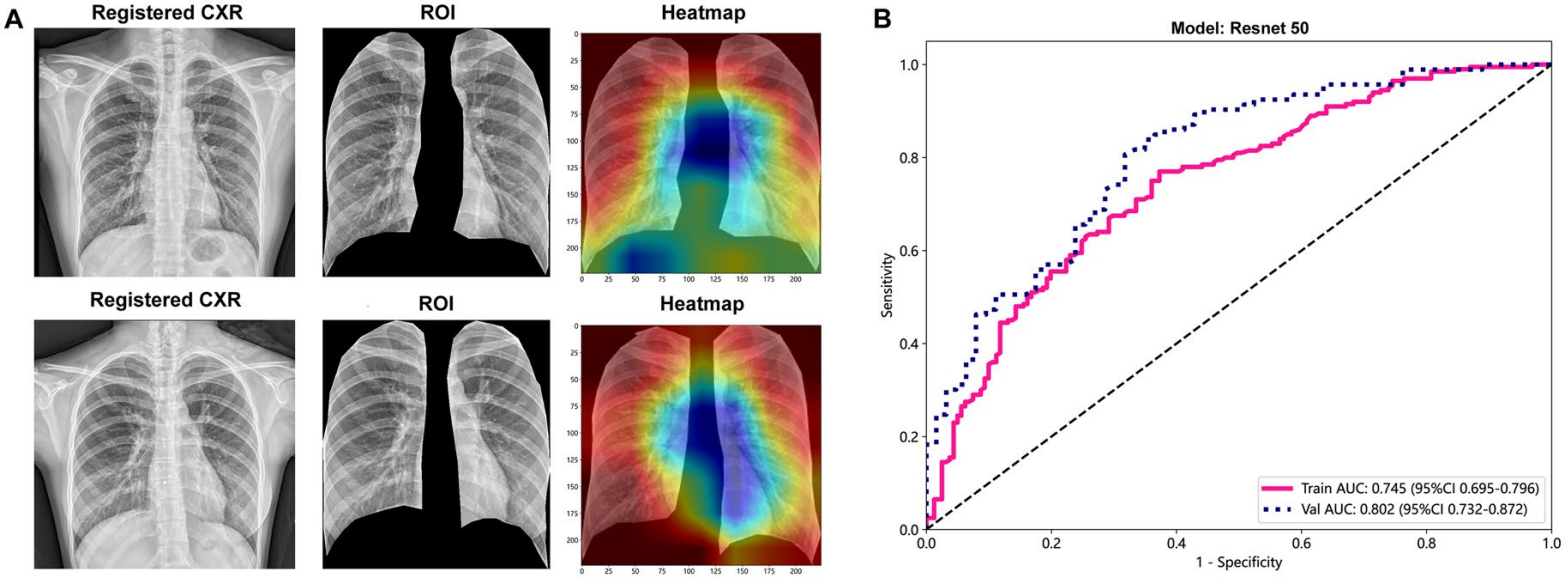
Interpretability and Performance of the deep learning model. (A) CNNs identifies features from the chest X-rays of COVID-19 patients that are associated with a higher likelihood of respiratory sequelae, marked as red hot spots in the heatmaps. (B) ROC of ResNet50 model. AUC: Area under the curve; CI: Confidence Interval; CXR: Chest X-ray; ROI: Region of interest.

Visualizing the model’s decision-making process is crucial for enhancing interpretability because DL models frequently have an opaque decision-making framework. Thus, we employed the Grad-CAM approach. Figure 4A illustrates the CXRs of two COVID-19 patients, the DL model’s primary input images and the corresponding heatmaps. The heatmap’s red areas indicate features strongly correlating with severe respiratory sequelae. Notably, the heatmaps show specific left lung areas as blue, which is likely due to the left lung’s cardiac notch. By utilizing the ResNet50 model, the DL model effectively distinguished between the two clusters, achieving an AUC of 0.745 (95% confidence interval [CI]: 0.695–0.796) and 0.802 (95% CI: 0.732–0.872) in the training and validation cohorts, respectively (Figure 4B). Supplementary Table 1 provides additional details on the DL model’s various performance indicators.

### 4.4. Construction and performance of the ensemble predictive model

**Figure 5.**
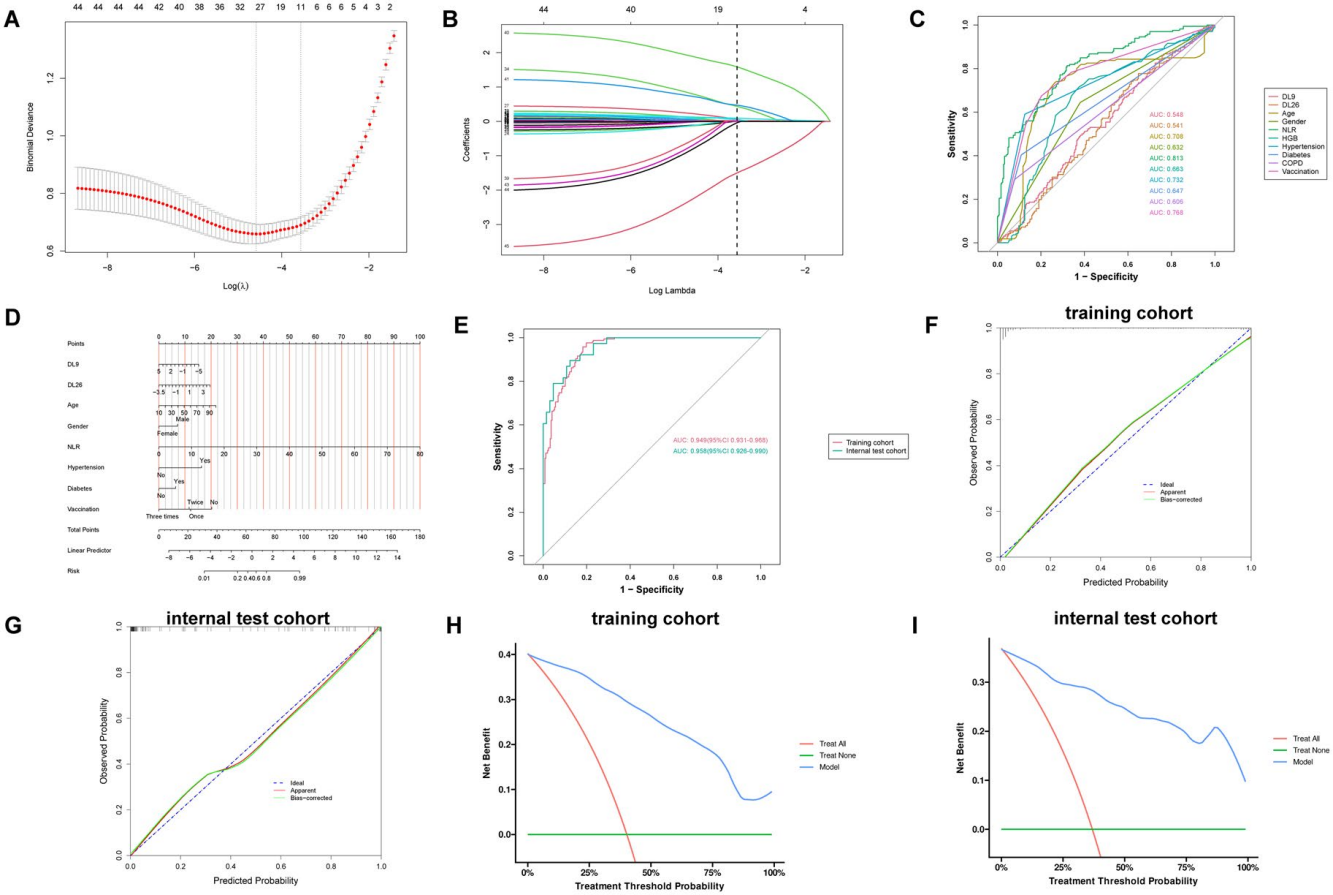
Construction and validation of the predictive model. (A) Cross-validation plot of the LASSO regression model; (B) Coefficient path plot of the LASSO regression model. (C) ROC curve analysis of the candidate diagnostic indicators; (D) Nomogram for predictive model; (E) ROC curves of the nomogram; (F) Calibration curve of the nomogram for the training cohort. (G) Calibration curve of the nomogram for the internal test cohort. (H) Decision curve analysis of the nomogram for the training cohort. (I) Decision curve analysis of the nomogram for the internal test cohort. AUC: Area under the curve; CI: Confidence Interval; NLR: Neutrophil-to-lymphocyte ratio; OR: Odds Ratio; HGB: haemoglobin.

To create a comprehensive dataset, 32 DL features were extracted from the fully connected layer within the CNNs and integrated with clinical data. LASSO regression analysis helped to filter all the training cohort variables of this dataset. The LASSO regression model’s cross-validation plot is shown in Figure 5A and B depicts the coefficient path plot. Ten variables comprised the most regularized and parsimonious model, with a cross-validated error within one standard error of the minimum. Figure 5C displays the ten variables’ ROC curves, each yielding AUC >0.5. Excluding other confounding factors, a multivariate logistic regression analysis was performed to determine whether these ten variables were independent predictors. Moreover, DL9, DL26, age, male gender, NLR, vaccination status, hypertension and diabetes history were significantly associated with severe respiratory sequelae (*p* < 0.05, Table 3). The final logistic model included eight independent predictors and helped in developing a user-friendly nomogram (Figure 5D). The nomogram’s AUCs in the different cohorts were 0.949 and 0.958, respectively (Figure 5E). In both training and internal validation cohorts, the calibration curves demonstrated excellent agreement between predicted and observed outcomes. For the training cohort, the calibration slope was 0.954 (Brier score = 0.087), while the internal validation cohort showed near-perfect calibration (slope = 1.005; Brier score = 0.083). The Hosmer-Lemeshow test indicated nonsignificant deviation in both cohorts (training: *p* = 0.885; validation: *p* = 0.751; Figure 5F-G). Decision curve analysis further revealed a superior net benefit of the nomogram compared to treat-all strategies across the clinically relevant threshold probability spectrum in both cohorts (Figure 5H-I). Supplementary Table 2 provides additional details regarding the predictive model’s performance indicators.

**Table 3.** Results of multivariate logistic regression for training cohort.

Characteristic	N	Event N	OR	95% CI	p-value
DL9	413	166	0.80	0.64, 0.99	0.045
DL26	413	166	1.47	1.05, 2.10	0.028
Age	413	166	1.03	1.02, 1.05	<0.001
Gender					
Female	212	59	—	—	
Male	201	107	2.76	1.39, 5.62	0.004
NLR	413	166	1.19	1.14, 1.26	<0.001
HGB	413	166	0.99	0.97, 1.00	0.051
Hypertension					
No	284	68	—	—	
Yes	129	98	9.77	4.56, 22.17	<0.001
Diabetes					
No	319	99	—	—	
Yes	94	67	2.30	1.03, 5.23	0.043
COPD					
No	346	118	—	—	
Yes	67	48	1.06	0.41, 2.73	0.907
Vaccination					
No	122	90	—	—	
Once	40	22	0.29	0.09, 0.87	0.028
Twice	52	18	0.25	0.08, 0.73	0.013
Three times	199	36	0.06	0.02, 0.13	<0.001

CI: Confidence Interval; NLR: Neutrophil-to-lymphocyte ratio; OR: Odds Ratio; HGB: haemoglobin.

In summary, the predictive model in this study effectively integrates complex CXRs and clinical data, offering a comprehensive approach to risk stratification. The use of LASSO regression ensures that only the most relevant and parsimonious predictors are included, enhancing the model’s interpretability and reducing the risk of overfitting. However, as the study is based on data from a single-center retrospective analysis and lacks external validation, its generalizability to broader populations remains uncertain. Future studies should focus on multi-center prospective research to assess the model’s applicability in diverse settings and investigate whether incorporating additional factors or larger datasets could enhance its predictive accuracy.

## 5. Discussion

### 5.1. Clinical significance of the AI-based predictive model

The integration of AI into clinical medicine has revolutionized the outlook towards disease diagnosis and prognosis. Thus, AI tools like UMLAs and CNNs are extensively utilized to enhance predictive accuracy in clinical scenarios. Therefore, we have created an AI-based predictive model for early identification of severe respiratory sequelae in COVID-19 patients. By applying the K-means algorithm and ResNet50, our model has demonstrated a significant potential in stratifying patients based on their risk levels. This risk stratification enables a paradigm shift from reactive care to proactive long-term management for COVID-19 survivors.

Using UMLAs to uncover novel subtypes can help construct accurate predictive models and promote precision medicine in the context of this heterogeneous disease. Zheyu Wang et al. utilized UMLAs to develop a risk score for Alzheimer’s disease progression [23]. Similarly, Demanse et al. identified clinical phenotypes within an osteoarthritis initiative database by using UMLAs, thereby enhancing patient classification and understanding [24]. Hence, clinicians can effectively impart customized interventions, improve patient outcomes and optimize the use of healthcare resources by identifying high-risk patients. In the context of COVID-19, identifying high-risk individuals during the convalescent phase could facilitate targeted follow-up strategies, including scheduled pulmonary function tests and early referral to pulmonary rehabilitation programs, even for those with initially mild presentations.

Radiomics extracts subvisual quantitative features from medical images, like intensity, texture and shape, to reveal underlying biological characteristics [25]. Unlike traditional radiomics models, DL models are better at grasping more representative features and are less prone to overfitting on limited training data, thereby enhancing their predictive performance [26]. Thus, integrating DL methods with clinical data has shown significant potential in boosting the model’s predictive power. The ability of DL to analyze complex datasets can also identify subtle patterns that could have been overlooked by traditional statistical methods. We used ResNet50 to perform DL analysis on COVID-19 patients’ CXRs for extracting 32 DL features. Following LASSO regression feature selection and multivariate logistic regression analysis, we identified two independent predictors, DL9 and DL26. Specifically, DL9 and DL26 correspond to feature maps obtained from layers 9 and 26 of the ResNet-50 model, respectively. These layers are pivotal in forming the deep representations that allow the model to distinguish between subtle variations in the CXR images related to the progression of COVID-19 and its associated respiratory sequelae.

DL9 captures low-level features, such as edges, simple textures and basic anatomical shapes in the lung fields. These features are crucial for the initial identification of lung structures and their potential alterations. In contrast, DL26 represents high-level, abstract features that capture more complex patterns, likely associated with pathological changes in lung structure. Together, these DL features underscore the importance of multi-level feature extraction in deep learning models. By representing both low- and high-level aspects of the image, they enable the model to develop a robust understanding of subtle variations in lung conditions. By using these image features in combination with clinical data, our predictive model achieves a higher level of precision in identifying high-risk patients.

Unlike traditional statistical approaches, AI models excel at handling multi-dimensional data, identifying subtle features in both images and clinical data and minimizing overfitting, even with limited training datasets. In the present study, the inclusion of DL features introduces novel, imaging-derived markers that may capture subtle structural or functional lung changes not detectable by traditional clinical assessments. This integration of ‘visible’ clinical factors and ‘invisible’ imaging signatures enhances the model’s ability to identify at-risk patients early, even before overt symptoms emerge. Notably, our cohort was sourced from a fever clinic managing mild-to-moderate COVID-19 cases, resulting in a population with lower initial clinical severity. This is evidenced by the acute-phase CXR findings, where nearly half of the patients presented with no radiographic abnormalities and the predominant observations were mild opacities. The model’s capacity to effectively stratify risk in such a population strongly indicates its ability to detect subtle, preclinical signatures of susceptibility to long-term sequelae, rather than simply mirroring the severity of acute pneumonia. This capability is particularly valuable for identifying the ‘high-risk but non-hypoxic’ population that might otherwise be overlooked in standard acute care protocols focused primarily on hypoxic respiratory failure.

The final nomogram exhibits exceptional performance across key metrics. The high AUCs indicate superior discriminative ability. This level of accuracy is critical for clinical utility. Equally important, the calibration curves and low Brier scores demonstrate that the model’s predictions align closely with observed outcomes. The non-significant Hosmer-Lemeshow tests further confirm this calibration, reinforcing the model’s reliability in estimating risk across different patient subgroups. In addition, to avoid overfitting, we standardized clinical variables and CXRs to reduce variability. The data were split 8:2 into training and validation cohorts. The ResNet50 model used early stopping with L2 regularization, while LASSO regression selected key features for the ensemble model to ensure parsimony. Consistent performance across cohorts confirmed robust generalization without overfitting.

While we acknowledge that current COVID-19 treatments primarily target acute mortality reduction rather than preventing long-term sequelae, the clinical value of our model lies in enabling structured post-discharge management. It provides a framework for implementing closer monitoring and early rehabilitation for high-risk individuals and could serve as a stratification tool for future clinical trials evaluating interventions aimed at preventing chronic pulmonary complications.

However, AI-based predictive models have certain limitations. These models often function as ‘black boxes,’ necessitating supplementary techniques, such as Grad-CAM, to improve interpretability. Additionally, the implementation of AI solutions demands significant computational resources and expertise, which may not be readily accessible in all clinical settings.

### 5.2. Risk factors related to severe respiratory sequelae in COVID-19 patients

Analyzing the risk factors associated with severe respiratory sequelae in COVID-19 patients highlights the model’s clinical relevance. We identified several key risk factors, including DL9, DL26, age, male gender, NLR, vaccination status, hypertension and diabetes. However, the evidence for an increased incidence of other chronic respiratory diseases following COVID-19 is limited [27,28]. Moreover, there is a growing awareness regarding the heightened risks of pulmonary fibrosis and pulmonary embolism in COVID-19 survivors, which could contribute to respiratory symptoms like breathlessness, erratic breathing and persistent cough [29].

The association between COVID-19 and acute thromboembolic disease is well established. The pulmonary embolism mechanisms following COVID-19 are complicated and involve a complex interplay of endothelial injury, hypercoagulability, blood flow stasis, impaired fibrinolysis and direct viral effects [30]. In the context of COVID-19, significant risk factors for venous thromboembolic complications are male predilection, older age, mechanical ventilation, elevated C-reactive protein and increased D-dimer levels [31,32].

Organizing pneumonia is a common radiological pattern in COVID-19 and shows fibrotic remodeling in a few cases [33,34]. Persistent radiological abnormalities after one year post-COVID-19 pneumonia range from limited ground-glass opacity and subpleural reticulation to extensive ground-glass opacity, traction bronchiectasis and honeycomb pattern [35]. Furthermore, severe COVID-19 and pulmonary fibrosis are more prevalent in men and the elderly, as well as associated with type 2 diabetes and hypertension [36,37]. Thus, these findings are consistent with our results.

Although numerous studies have established associations between pulmonary fibrosis, pulmonary embolism and the immune response during the acute COVID-19 phase, direct evidence linking NLR to respiratory sequelae in COVID-19 patients is lacking to date. Patients in the early post-acute COVID-19 phase displayed elevated levels of neutrophil extracellular traps (NETs), which exogenously stimulate epithelial cell fibrogenesis [38,39]. Furthermore, the hyperinflammatory immune response during the acute COVID-19 phase predisposes patients to microvascular thrombosis [40]. Thus, an intensified inflammatory response in the acute phase might significantly help in the development of pulmonary fibrosis and embolism in the post-COVID-19 period. NLR, a hematological parameter measuring the relative neutrophil and lymphocyte levels, reflects the body’s immune response to SARS-CoV-2 and indicates an enhanced inflammatory state. Therefore, elevated NLR levels might be a valuable tool for understanding the immune status and potential outcomes in COVID-19 patients [41]. We showed that elevated NLR might be an independent risk factor for severe respiratory sequelae in COVID-19 patients. Hence, monitoring NLR during follow-up visits could effectively help assess the risk of severe respiratory sequelae.

### 5.3. Challenges and future directions

While our findings demonstrate the potential of this model for early identification and intervention, several challenges must be addressed to optimize its clinical utility and long-term effectiveness.

One significant challenge is the threat to the model’s validity. Our data were derived from a single-center retrospective study, which inherently limits the external applicability of the model to diverse patient populations across different healthcare settings. Variability in demographics, comorbidities and clinical management practices may influence the model’s predictive accuracy in other populations. Beyond the issues of generalizability, the scope of acute-phase treatment data was also limited. Given that our cohort consisted primarily of mild-to-moderate cases from a fever clinic, the administration of specific therapeutics—such as antivirals and immunomodulators—was infrequent and exhibited insufficient variability for reliable statistical evaluation. Additionally, the retrospective nature of the dataset presents potential biases. The historical nature of the data may not adequately reflect evolving trends in COVID-19 treatment or emerging variants of the virus, which could lead to shifts in disease progression and patient outcomes. To address these limitations collectively and validate the model’s generalizability, multi-center prospective clinical studies remain an essential next step for further refinement and real-world verification of our predictive framework. Additionally, the retrospective nature of the dataset presents potential biases. The historical nature of the data may not adequately reflect evolving trends in COVID-19 treatment or emerging variants of the virus, which could lead to shifts in disease progression and patient outcomes. Furthermore, the risk of overfitting remains a concern, particularly when working with CNNs. While CNNs excel at extracting features from complex data, there is a possibility that the model has become too tailored to the specific dataset, limiting its ability to generalize to new, unseen cases.

Despite these challenges, there are several key directions for future research. First, external validation with multicentre datasets is essential to enhance the model’s generalizability and ensure that it can be applied effectively across a wide range of healthcare settings. Furthermore, to improve the model’s predictive accuracy, future studies could incorporate longitudinal data, allowing for the capture of temporal variations in patient health and respiratory outcomes. Real-time data integration could also enhance the model’s responsiveness, enabling clinicians to make more accurate and timely predictions based on current patient conditions.

However, larger datasets require more storage capacity, longer training times for the ensemble models and enhanced processing power to avoid bottlenecks in data curation, preprocessing and model retraining. Additionally, multi-center studies will necessitate distributed computing frameworks or cloud-based solutions to aggregate and analyze data across sites, further straining resource requirements. For successful clinical deployment, addressing computational resource limitations is paramount. This includes investing in GPU/TPU-enabled workstations, scalable cloud platforms and high-speed data storage systems, particularly in resource-constrained settings.

In conclusion, while the AI-driven predictive model developed in this study shows great promise, addressing these challenges and refining the model through validation, continuous learning and explainability will be crucial to its success in real-world clinical applications. By focusing on these areas, we aim to improve the early identification and management of COVID-19 patients at risk of severe respiratory sequelae, ultimately reducing the long-term health burden associated with the disease.

## 6. Conclusions

Our AI-driven predictive model demonstrates strong potential for the early identification of respiratory sequelae in COVID-19 patients. By applying the K-means algorithm to cluster patients based on clinical characteristics, in combination with feature extraction from chest X-rays using the ResNet-50 deep learning model, we accurately stratified patients by their risk of severe respiratory outcomes. However, to evaluate its performance in clinical settings, further validation using larger, independent datasets is essential to confirm the model’s reliability and generalizability across diverse populations.

## Supplementary Material

Supplementary Material.docx

## Data Availability

The original contributions presented in this study are available in the article/supplementary material. Further inquiries can be directed to the corresponding authors.
