# Supplementary material for: AI-driven prediction of severe respiratory sequelae in COVID-19 patients
Source: Ann Med. 2025 Dec 12;57(1):2598914. doi: 10.1080/07853890.2025.2598914 (PMC12704136; doi:10.1080/07853890.2025.2598914)
Supplement: Supplementary Material.docx [file IANN_A_2598914_SM6239.docx]

**Supplementary Material**

Supplementary table 1: Modeling results using convolutional neural networks

| Model Name | Acc | AUC | 95% CI | Sensitivity | Specificity | PPV | NPV | Precision | Recall | F1 | Threshold | Cohort |
| --- | --- | --- | --- | --- | --- | --- | --- | --- | --- | --- | --- | --- |
| Resnet50 | 0.701 | 0.745 | 0.6947-0.7960 | 0.760 | 0.627 | 0.717 | 0.678 | 0.717 | 0.760 | 0.738 | 0.490 | Train |
| Resnet50 | 0.756 | 0.802 | 0.7324-0.8720 | 0.828 | 0.651 | 0.778 | 0.719 | 0.778 | 0.828 | 0.802 | 0.419 | Test |

Acc: accuracy; AUC: area under the curve; CI: confidence interval; PPV: positive predictive value; NPV: negative predictive value.

Supplementary table 2: Classification accuracy for prediction at different risk cutoff points for the model in training cohort

| Risk score  threshold | Linear Predictor  Cutoff Point | Sensitivity  (%) | Specificity  (%) | PPV  (%) | NPV  (%) | Accuracy  (%) | Precision  (%) | Recall  (%) | F1 |
| --- | --- | --- | --- | --- | --- | --- | --- | --- | --- |
| ≥ 0% | -Inf | 100.0 | 0.0 | 40.2 |  | 40.2 | 40.2 | 100.0 | 0.573 |
| ≥ 10% | -2.1972246 | 98.8 | 69.2 | 68.3 | 98.8 | 81.1 | 68.3 | 98.8 | 0.808 |
| ≥ 20% | -1.3862944 | 98.2 | 75.7 | 73.1 | 98.4 | 84.7 | 73.1 | 98.2 | 0.838 |
| ≥ 30% | -0.8472979 | 93.4 | 83.4 | 79.1 | 94.9 | 87.4 | 79.1 | 93.4 | 0.856 |
| ≥ 40% | -0.4054651 | 89.2 | 87.0 | 82.2 | 92.3 | 87.9 | 82.2 | 89.2 | 0.855 |
| ≥ 50% | 0.0000000 | 84.3 | 89.1 | 83.8 | 89.4 | 87.2 | 83.8 | 84.3 | 0.841 |
| ≥ 60% | 0.4054651 | 78.9 | 92.7 | 87.9 | 86.7 | 87.2 | 87.9 | 78.9 | 0.832 |
| ≥ 70% | 0.8472979 | 70.5 | 94.7 | 90.0 | 82.7 | 85.0 | 90.0 | 70.5 | 0.791 |
| ≥ 80% | 1.3862944 | 62.0 | 96.0 | 91.2 | 79.0 | 82.3 | 91.2 | 62.0 | 0.738 |
| ≥ 90% | 2.1972246 | 47.0 | 96.8 | 90.7 | 73.1 | 76.8 | 90.7 | 47.0 | 0.619 |
| ≥ 100% | Inf | 0.0 | 100.0 |  | 59.8 | 59.8 |  | 0.0 |  |

PPV: positive predictive value; NPV: negative predictive value.
